# A dataset on topsoil salinization characteristics in the Tailan River Irrigation District on the northern margin of the Tarim Basin in Xinjiang

**DOI:** 10.1038/s41597-026-06977-y

**Published:** 2026-03-05

**Authors:** Qianxi Zhang, Miaomiao Gong, Wei Luo, Jingtong He, Jinhua Ding, Pingan Jiang

**Affiliations:** 1 College of Hydraulic and Civil Engineering, Xinjiang Agricultural University, No.311 Nongda East Road, Shayibake District, Urumqi, 830052 Xinjiang Uygur Autonomous Region China; 2 Technical Research Center of Xinjiang Hydro-Geotechnical and Structural Engineering, No.311 Nongda East Road, Shayibake District, Urumqi, 830052 Xinjiang Uygur Autonomous Region China; 3 Xinjiang Key Laboratory of Hydraulic Engineering Safety and Water Disaster Prevention, No.311 Nongda East Road, Shayibake District, Urumqi, 830052 Xinjiang Uygur Autonomous Region China; 4 College of Civil Engineering and Architecture, Xinjiang University, No. 777 Huarui Street, Shuimogou District, Urumqi, 830018 Xinjiang Uygur Autonomous Region China; 5 Xinjiang Agricultural University, No.311 Nongda East Road, Shayibake District, Urumqi, 830052 Xinjiang Uygur Autonomous Region China

**Keywords:** Geochemistry, Agriculture

## Abstract

Soil salinization is a critical barrier to sustainable agriculture and ecological stability in arid and semi-arid regions. The Tailan River Irrigation District in southern Xinjiang is severely affected by salinization, with a two-decade absence of comprehensive soil salinity survey data hampering effective saline-alkali land management. This study established 164 sampling points in the district via equal-interval zoned sampling in ArcGIS, collecting 0–120 cm topsoil samples at seven depths on April 20–21, 2024 in accordance with NY/T 395–2012. Soil samples were pretreated for EC measurement (1:5 soil/water ratio), and physico-chemical properties, including pH, electrical conductivity (EC), moisture content (MC), total soluble salt content (TSS) and eight salt ion content, were analyzed per national agricultural and environmental standards. After rigorous screening, 118 valid sampling points and 807 soil data records were used to construct a comprehensive dataset that clarifies the spatial distribution of soil salinity, moisture and EC in the district, providing a vital foundation for saline-alkali land improvement, crop layout optimization and the formulation of relevant agricultural policies.

## Background & Summary

Soil salinization presents a significant environmental challenge globally, threatening agricultural productivity and ecological health^1–5^. This issue has become a widespread ecological concern, garnering attention from nations around the world^6–8^. The Food and Agriculture Organization (FAO)^9^ (2026) reports that salinity-affected soils now exceed 1 billion hectares worldwide, affecting nearly one-fifth of the world’s arable land and one-third of all irrigated land^10,11^. Soil salinity adversely impacts seed germination, agricultural yields, and the quality of both soil and water. This phenomenon is particularly acute in arid and semi-arid regions, where it accelerates the degradation of arable land and reduces overall land quality^12–14^.

In China, the area of arable saline-alkali land encompasses approximately 36.7 million hectares, which accounts for about one-third of the nation’s total arable land area^15^. Xinjiang, a typical arid and semi-arid region in northwestern China, contains the largest and most extensive distribution of saline-alkali land in the country^16^. The Tailan River Irrigation District, a prominent example of artificial oasis irrigation districts in southern Xinjiang, is located in Wensu County, Aksu Prefecture. This region experiences an annual average precipitation of 62.1 mm, while the annual average evaporation exceeds 2000 mm since 1950. This area is rich in resources, including grain, cotton, oilseeds, livestock products, and fruits^17^. The region primarily relies on surface water from the Tailan River and its tributaries^18^. However, significant annual variability in the water volume of the Tailan River, characterized by marked differences between flood and dry seasons and an uneven annual distribution, combined with the current absence of regulating reservoirs, leads to severe spring droughts and frequent summer flood hazards. Monitoring data from groundwater observation wells reveal a significant year-on-year decline in groundwater levels within the Tailan River Irrigation Area. This reduction results from two primary factors. First, the construction of numerous seepage-control canals during extensive water conservancy projects has decreased surface water infiltration and recharge. Second, concentrated groundwater extraction in areas reliant solely on well irrigation has exacerbated the decline of the water table.

Although improvements in water conservancy infrastructure have occurred over the years, the issue of soil salinization persists. In the past two decades, the promotion of under-film drip irrigation technology in the district has resulted in the emergence of secondary soil salinization. According to the survey results from the 2007 Saline-Alkali Land Improvement Plan of Wensu County, Within the surveyed area of 70,666 hectares in the Tailan River Irrigation District, 9,526 hectares were affected by salinization, accounting for 13.5%, of which 4,819 hectares were moderately to severely salinized. However, the absence of comprehensive surveys on soil salinization within the district has resulted in an unclear distribution of salinity across arable land in various regions. This ambiguity complicates the implementation of targeted measures for saline-alkali land improvement. Although recent advancements in remote sensing technology have improved the identification of soil types and characteristics over extensive areas, on-site investigations and sampling for soil salinity measurement remain essential for accurately assessing soil properties and calibrating the results of remote sensing interpretations^19^.

This study examines soil salinization in the Tailan River Irrigation District. By integrating on-site sampling, laboratory experiments, and statistical data analysis, we establish a comprehensive dataset that details the physical and chemical properties of the soil within the irrigation district and delineates the spatial distribution of soil salinity. Analyzing total soluble salt content (TSS), salt ion content, moisture content (MC), pH value, and electrical conductivity (EC) forms the basis for efforts to mitigate salinization. The above core indicators directly influence the scientific validity, specificity, and operability of improvement plans, which are essential for achieving the sustainable use of soil and water resources in the irrigation area and for enhancing agricultural productivity.

## Methods

### Study area

The study area (80°21′44′-81°10′14″E, 40°41′41″-42°15′13″N, China GCJ-02 Coordinate System developed by the National Administration of Surveying, Mapping and Geo-information of China to derive from the WGS84 coordinate system through encryption processing for the collection and utilization of geographic data within Chinese borders) is located in the Tailan River Irrigation District in southeastern Wensu County, Akesu Prefecture. It lies at the northern edge of the Xinjiang Tarim Basin, approximately 12 kilometers from Wensu County. The terrain exhibits a gradual slope descending from north to south and from west to east. The entire district is divided into two distinct regions: the northern mountainous area and the southern alluvial proluvial plain.

The Tailan River Irrigation Area exhibits a continental arid climate typical of the northern temperate zone. Seasonal variations are pronounced, characterized by long winters and springs, short summers and autumns, and a mild climate with significant diurnal temperature fluctuations. The multi-year average precipitation measures 62.1 mm, predominantly occurring from June to August, while winter experiences minimal rainfall. The maximum daily precipitation recorded is 48 mm since 1950. The mean annual temperature is 9.8 °C, with July averaging 31 °C and January averaging −14.7 °C. Extreme temperatures range from 40.7 °C to −27.6 °C, resulting in an annual temperature differential of 45.7 °C. The annual average wind speed is 1.72 m/s, with a maximum average wind speed reaching 15 m/s, primarily originating from the west. During the spring sowing period, from March to May, the average relative humidity is only 58%.

**Fig. 1:**
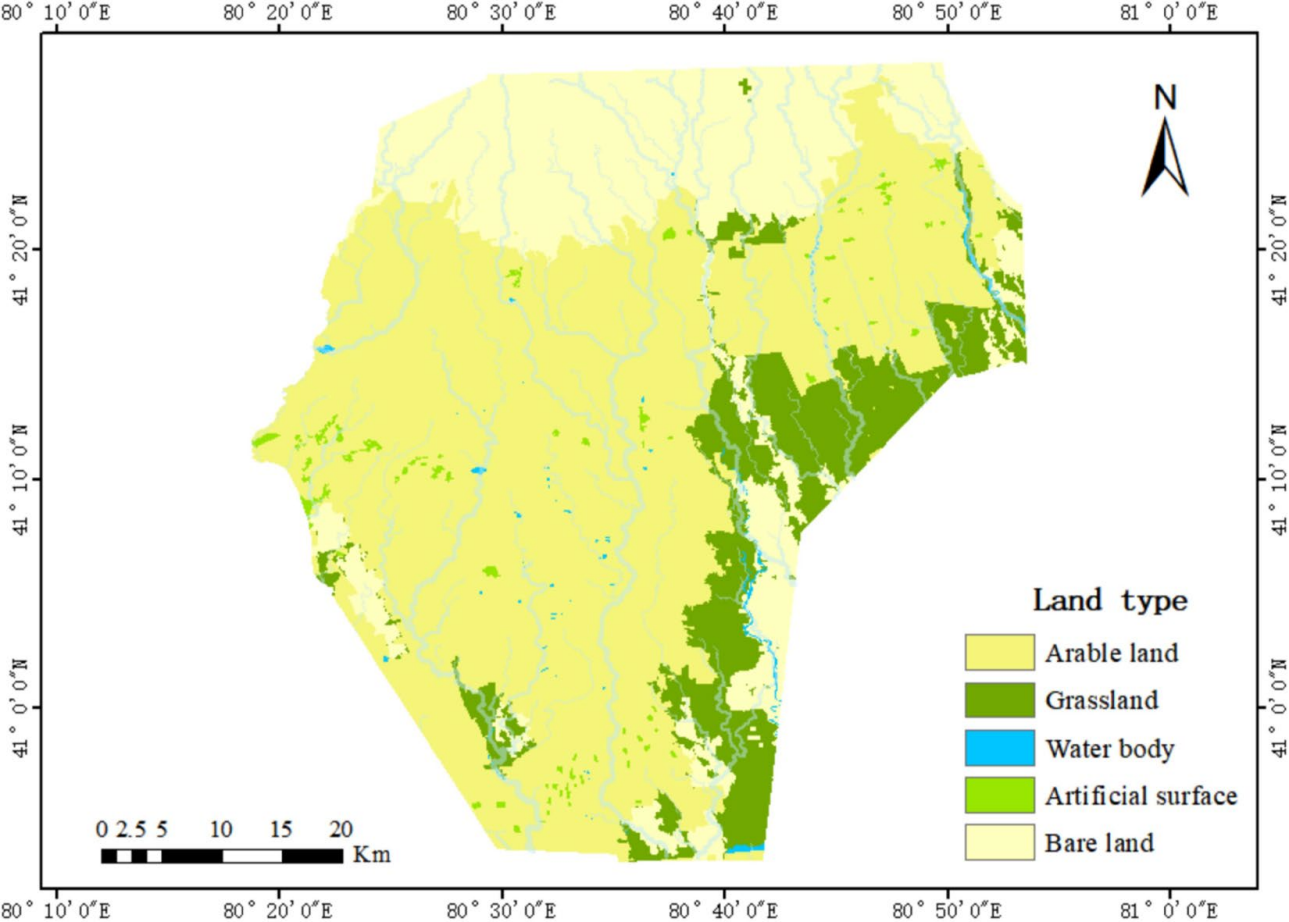
Map of Land Use Types in the Tailan River Irrigation Area. It presents the land type and river distribution of the Tailan River Irrigation Area in 2023, derived from GlobeLand30 global land cover data and digital elevation model (DEM) data from the Geospatial Data Cloud. The color scheme in the figure indicates that dark yellow represents cultivated land, dark green denotes grassland, blue signifies water bodies, light green indicates artificial ground surfaces, and light yellow represents bare land.

**Fig. 2:**
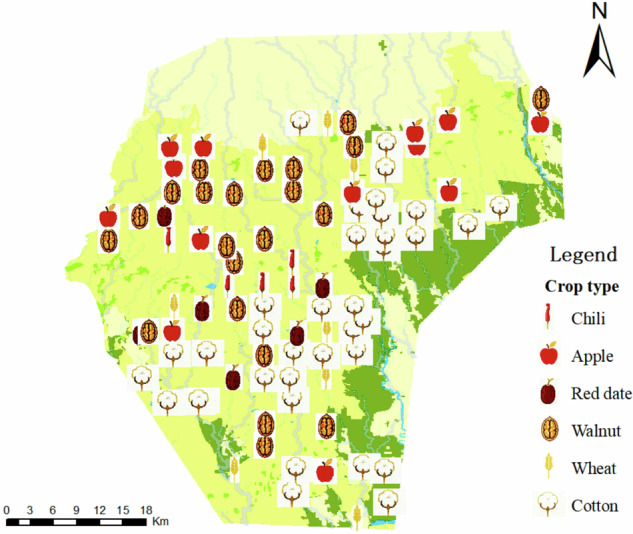
Map of Crop Types in the Study Area. It presents field sampling records that illustrate the distribution of the main crops cultivated in the Tailan River Irrigation Area.

Relevant data indicate that the large-scale and systematic development of water conservancy projects and farmland reclamation in the Tailan River Irrigation Area began primarily in the 1960s. This suggests that the continuous cropping history of many cultivated fields within the irrigation area has likely exceeded 60 years. Figure 1 presents the 2023 land type and river distribution of the Tailan River Irrigation Area, generated using GlobeLand30 global land cover data and digital elevation model (DEM) data from the Geospatial Data Cloud. The map shows that cultivated land constitutes 61.99% of the area of the Tailai River Irrigation District, grassland comprises 12.78%, water bodies account for 0.45%, artificial surfaces represent 0.80%, and bare land encompasses 23.98%. The field sampling records illustrate the distribution of the main crops cultivated in the Tailan River Irrigation Area, as shown in Fig. 2.

In the Tailan River Irrigation Area, conventional irrigation and drip irrigation serve as the primary methods of irrigation. The total irrigated area, excluding multiple cropping, encompasses 62,313 hectares. High-efficiency water-saving irrigation accounts for 19,813 hectares, yielding a water-saving irrigation rate of 31.8%. The southern section of the irrigation area is mainly allocated for cotton cultivation, where drip irrigation is the predominant method. This region is currently undergoing a transition from traditional flood irrigation to more efficient water-saving drip irrigation.

### Field sampling

**Fig. 3:**
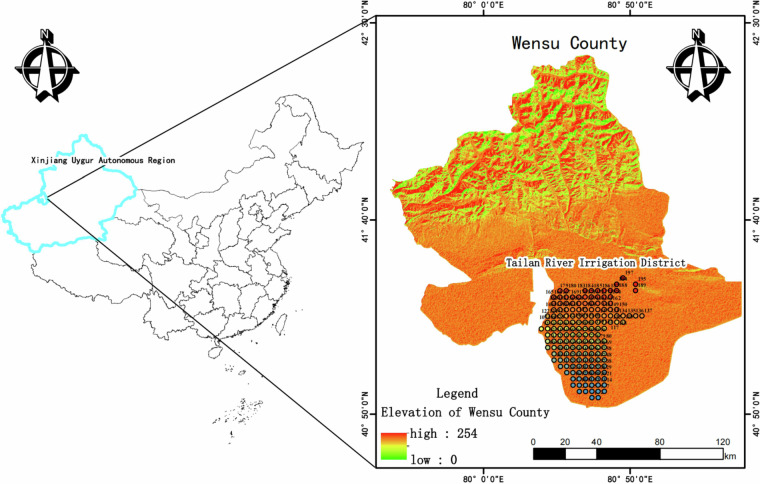
Layout map of sampling points. It presents that an equal-interval zoned sampling approach was implemented in the Tailan River Irrigation District using ArcGIS, encompassing a total of 164 sampling points.

In the Tailan River Irrigation District, an equal-interval zoned sampling approach was implemented using ArcGIS, encompassing a total of 164 sampling points (refer to Fig. 3). The distance between each sampling point ranged from 2,000 m to 2,500 m. Following *Technical Specifications for Monitoring of Farmland Soil Environmental Quality* (NY/T 395–2012)^20^, seven soil layers (0–5 cm, 5–20 cm, 20–40 cm, 40–60 cm, 60–80 cm, 80–100 cm, 100–120 cm) within the 0–120 cm depth were sampled at each site. A single soil sample was obtained from each layer, placed in labeled sample bags, sealed, and assigned serial numbers. The samples were subsequently transported to the laboratory quickly for analysis of moisture content (MC) in 3 days after sampling, and then for total suspended solids (TSS), salt ions, pH, and electrical conductivity (EC).

### Experimental methods

#### Preparation of leachate

The soil samples were pretreated following the standard operating procedure for measuring soil electrical conductivity (soil/water, 1:5) recommended by the Food and Agriculture Organization of the United Nations (FAO). An air-dried soil sample weighing 60 g passes through a 2 mm sieve with an accuracy of 0.01 g and is placed in a wide-mouth bottle. Pure water is then added at a soil-to-water ratio of 1:5, and the mixture is agitated vigorously for 3 minutes. Wet filter paper, moistened with pure water, is affixed to the bottom of a funnel for the filtration process. If the filtrate appears turbid, it is re-filtered. Should turbidity persist after repeated filtration, a centrifuge may be employed for further separation. The resulting clear filtrate is designated as the soil leachate and is stored in a narrow-mouth bottle for subsequent analysis.

#### pH Value determination test

According to the Chinese agricultural industry standard, *soil testing Part2: method for determination of soil pH (NY/T 1121.2-2006)*^21^, at least two different types of pH standard buffer solutions are required to calibrate the pH meter (Puchun PHS-3). The calibration process begins with the pH 6.86 (25 °C) standard buffer solution. Following this initial calibration, either the pH 4.01 (25 °C) or the pH 9.18 (25 °C) standard buffer solution is used for further calibration.

The sample temperature must be maintained at (25 ± 1) °C, ensuring that the temperature difference between the sample and the standard buffer solution does not exceed 2 °C. Immerse the electrode into the sample suspension, positioning the probe at a vertical depth of 1/3 to 2/3 below the liquid surface, and gently agitate the sample. Once the reading stabilizes, record the pH value. After measuring each sample, rinse the electrode promptly with pure water, blot any excess water from the electrode’s exterior using filter paper, and then proceed to measure the subsequent sample.

#### Moisture content test (oven-drying method)

According to the Chinese national standard, *Standard for Geotechnical Test Method (GB/T50123-2019)*^22^, take 15–30 g of a representative soil sample; for organic or sandy soil, take 50 g. Place the sample in a weighing dish, cover it with a lid, and weigh the total mass of the dish plus the moist soil, accurate to 0.01 g. Open the weighing dish and place it in an oven to dry at a constant temperature until it reaches a constant mass. The soil samples are preliminarily judged to be free of organic matter based on physical characteristics such as color, texture, and structure. Therefore, the drying temperature is set at 105–110 °C. The drying duration must be at least 8 hours for clay and silt and a minimum of 6 hours for sandy soil. After drying, remove the weighing dish from the oven, seal it with the lid, and cool it to room temperature in a desiccator. Then, weigh the dish along with the dry soil, ensuring accuracy to 0.01 g.

Calculate the soil MC using the following formula, rounding the result to one decimal place (0.1%).

1 $$MC=\left(\frac{{m}_{0}}{{m}_{{\rm{d}}}}-1\right)\times 100$$

where, *m*_0_ represents the mass of moist soil(g), and *m*_d_ represents the mass of dry soil(g).

#### Electrical conductivity (EC) determination test

According to the Chinese national environmental protection standards, *Soil quality—Determination of conductivity—Electrode method (HJ 802-2016)*^23^, KCl solution serves as the preferred reference material for calibrating conductivity meters. The Shanghai Leici DDS-11A conductivity meter is utilized for this purpose. Prior to calibration, it is essential to verify the instrument’s condition and prepare the necessary calibration tools. The electrode is then immersed in the calibration solution, ensuring consistent contact for 2-3 minutes. The conductivity meter is adjusted according to the specifications to complete the calibration process. After calibration, the electrode is placed into a narrow-mouth bottle containing the leachate, and data is recorded once the conductivity meter indicates a stable reading.

#### Determination of total soluble salts (gravimetric method)

According to the Chinese agricultural industry standard *soil testing Part16:method for determination of total water-soluble salt (NY/T 1121.16-2006)*^24^, using a pipette, draw 50 mL to 100 mL of the leachate and transfer it into a pre-weighed evaporating dish that has been weighed to a constant mass. Subsequently, place the dish on a water bath to evaporate the leachate until dryness is achieved. The appearance of a yellowish-brown color in the evaporated residue indicates the presence of organic matter. To remove the organic matter, add a small amount of 15% hydrogen peroxide (H₂O₂) and continue heating on the water bath, repeating this treatment until the residue turns white. Afterward, place the evaporating dish in an oven and dry it at 105 °C to 110 °C for a duration of 4 to 8 hours. Once dried, remove the dish and allow it to cool in a desiccator. Finally, use an electronic balance to measure the total mass of the evaporating dish along with the sample, repeating this weighing process until the difference between two consecutive measurements is no greater than 0.0001 g.

If the evaporated residue of the leachate contains a significant amount of crystal water, the measured concentration of total soluble salts may exceed the actual value. In such cases, two evaporating dishes are utilized: 50 mL of the leachate is added to one dish, while 50 mL of pure water is added to the other. An equal volume of 2% sodium carbonate (Na₂CO₃) solution is then introduced to each dish, ensuring thorough mixing. The previously outlined procedures are followed, with the drying temperature adjusted to 180 °C. The concentration of total soluble salts (TSS) in grams per kilogram (g/kg) is calculated using the following formula:

2 $${\rm{TSS}}=\frac{({m}_{{\rm{mz}}}-{m}_{m})\frac{{V}_{{\rm{w}}}}{{V}_{{\rm{x1}}}}}{{m}_{{\rm{d}}}\times {10}^{-3}}$$

Where *m*_mz_ denotes the mass of the evaporating dish plus the dried residue (g), *m*_m_ indicates the mass of the evaporating dish (g), *V*_w_ represents the volume of pure water added to prepare the leachate (mL), *V*_x1_ signifies the volume of the leachate drawn (mL), and md refers to the mass of the dry soil used to prepare the leachate (g).

The test indicators, implementation standards, test methods, and instruments/equipment are presented in Table 1.

**Table 1:** test indicators, implementation standards, test methods, and equipment.

Test indicators	Implementation standards	Test methods	Equipment
pH	NY/T 1121.2-2006^21^	potentiometric method	PHS-3
Electrical Conductivity EC (mS/cm)	HJ 802-2016^23^	conductometric method	DDS-11A
Moisture Content MC (%)	GB/T 50123-2019^22^	oven-drying method	aluminum dish, oven
Total Soluble Salts TSS (g/kg)	NY/T 1121.16-2006^24^	gravimetric method	evaporating dish, water bath, oven
${{\rm{Cl}}}^{-}$ (g/kg)	NY/T 1121.17-2006^27^	Silver nitrate titration method	Buret, beaker, erlenmeyer flask
${{\rm{SO}}}_{4}^{2-}$ (g/kg)	NY/T 1121.18-2006^28^	EDTA Indirect Titration Method	Buret, beaker, erlenmeyer flask

## Data Records

Following data accuracy calibration and screening (see “*Technical Validation”* section), this dataset comprises soil physical and chemical parameters from 118 sampling points within the Tailan River Irrigation District, collected between April 20 and 21, 2024. The metadata includes the sample point name, sample point location (longitude and latitude), pH, EC (unit: mS/cm), MC (unit: %), TSS (unit: g/kg), and the contents of eight major ions (Cl^−^, SO_4_^2-^, CO_3_^2-^, HCO_3_^-^, Na^+^, K^+^, Ca^2+^, Mg^2+^, unit: g/kg).

The data files are freely accessible at the figshare link: 10.6084/m9.figshare.30084454^25^. This dataset is provided in XLSX format. One Excel file contains three data sheets, detailed as follows: Sheet 1 includes a total of 807 data entries, covering columns such as Soil Sample Number (SSN), EC, pH, MC, TSS, and the eight ion contents. The SSN 2-5 indicates that the soil sample was collected from sampling point 2, with a depth range of 0 to 5 cm, while the SSN 4-20 indicates that the soil sample was collected from sampling point 4, with a depth range of 5 to 20 cm. Sheet 2 contains 118 data entries, which provide longitude and latitude information for the 118 sampling points. Sheet 3 details the metadata information in Sheets 1 and 2, including definitions, formats, and collection rules for each data field, as shown in Table 2. The “/“ in the data file indicates no data.

**Table 2:** Variables and their definitions in the dataset.

Field Name	Variable Code Name	Data Type	Dimension	Explanation
Sampling Point	SP	Textual Data	None	in-site sampling point
Soil Sample Number	SSN	Textual Data	None	Sample Point and Corresponding Soil Sampling Depth, for example, 2-5 represent that the soil sample is collected from sampling point 2, with a depth range of 0 to 5 cm
Moisture Content	MC	Floating-Point Type	None	Unit: %
pH	pH	Floating-Point Type	None	None
Electrical Conductivity	EC	Floating-Point Type	M⁻¹L⁻³T³I²	Unit: mS/cm
Total Amount of Soluble Salts	TSS	Floating-Point Type	None	Unit: g/kg
${{\rm{Cl}}}^{-}$	${{\rm{Cl}}}^{-}$	Floating-Point Type	None	Unit: g/kg
${{\rm{SO}}}_{4}^{2-}$	${{\rm{SO}}}_{4}^{2-}$	Floating-Point Type	None	Unit: g/kg
${{\rm{C}}{\rm{O}}}_{3}^{2-}$	${{\rm{CO}}}_{3}^{2-}$	Floating-Point Type	None	Unit: g/kg
HCO_3_^-^	HCO_3_^-^	Floating-Point Type	None	Unit: g/kg
K^+^	K^+^	Floating-Point Type	None	Unit: g/kg
Na^+^	Na^+^	Floating-Point Type	None	Unit: g/kg
Ca^2+^	Ca^2+^	Floating-Point Type	None	Unit: g/kg
Mg^2+^	Mg^2+^	Floating-Point Type	None	Unit: g/kg
Longitude	Lng	Floating-Point Type	None	The GCJ-02 Coordinate System, a geographic information system coordinate system established by the National Bureau of Surveying and Mapping of China, is derived from the WGS84 coordinate system through encryption processing for the collection and utilization of geographic data within Chinese borders.
Latitude	Lat	Floating-Point Type	None	

## Data Overview

In the study of soil salinization, total soluble salt content TSS, electrical conductivity EC, pH, and moisture content MC are key indicators for assessing salinization levels. These factors are interconnected, directly or indirectly, influencing each other and collectively determining the type, extent, and trends of salinization. TSS represents the cumulative concentration of soluble salt ions (e.g., Na^+^, K^+^, Ca²^+^, Mg²^+^, Cl^−^, SO₄²^-^, and HCO₃^-^) in the soil, serving as a fundamental indicator of salinization extent with a significant positive correlation. EC indicates the soil water solution’s ability to conduct electric current, directly related to the concentration and mobility of salt ions, showing a strong positive correlation with salinization degree and serving as a rapid, convenient indicator. Increased salinity often accompanies higher pH (alkalization), causing combined salt-alkali stress that is more detrimental to plants, with pH also impacting salt ion composition.

This soil dataset reveals the spatial distribution characteristics of soil physical and chemical properties in irrigation areas. First, ArcGIS software is used to verify and correct the normality of the measurement-point data. The optimal model and parameters are obtained by fitting the semivariogram. Then, spatial interpolation of each characteristic index is performed using ordinary Kriging to produce the spatial-distribution map for each index.

**Fig. 4:**
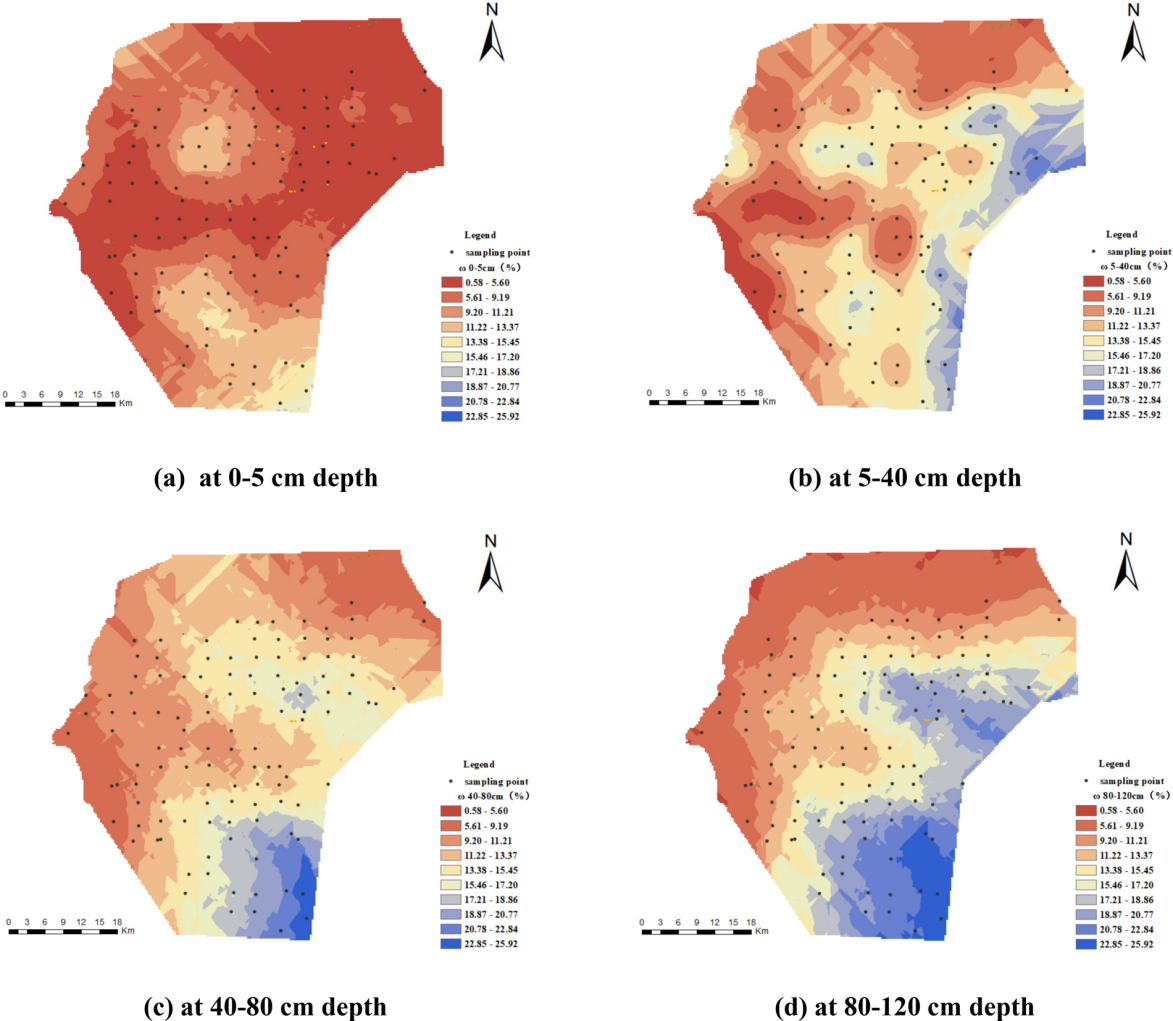
Spatial distribution of soil moisture content (MC) (%). (**a**) represents MC at 0-5 cm depth, (**b**) represents MC at 5-40 cm depth, (**c**) represents MC at 40-80 cm depth, and (**d**) represents MC at 80–120 cm depth.

Figure 4 demonstrates the spatial distribution of soil MC across the horizontal plane, showing a gradual increase from the northwest to the southeast. High MC regions above 15%(light blue) are mainly in the southeast across all depths (5–40 cm, 40–80 cm, 80–120 cm), while low MC areas (red and orange) are prevalent in the northwestern and northern regions. The surface soil MC remains below 5%, dropping even lower in the northern desert area. With increasing depth, MC rises, stabilizing below 40 cm. In the southeast, MC consistently exceeds 15%, with some areas reaching up to 30%.

**Fig. 5:**
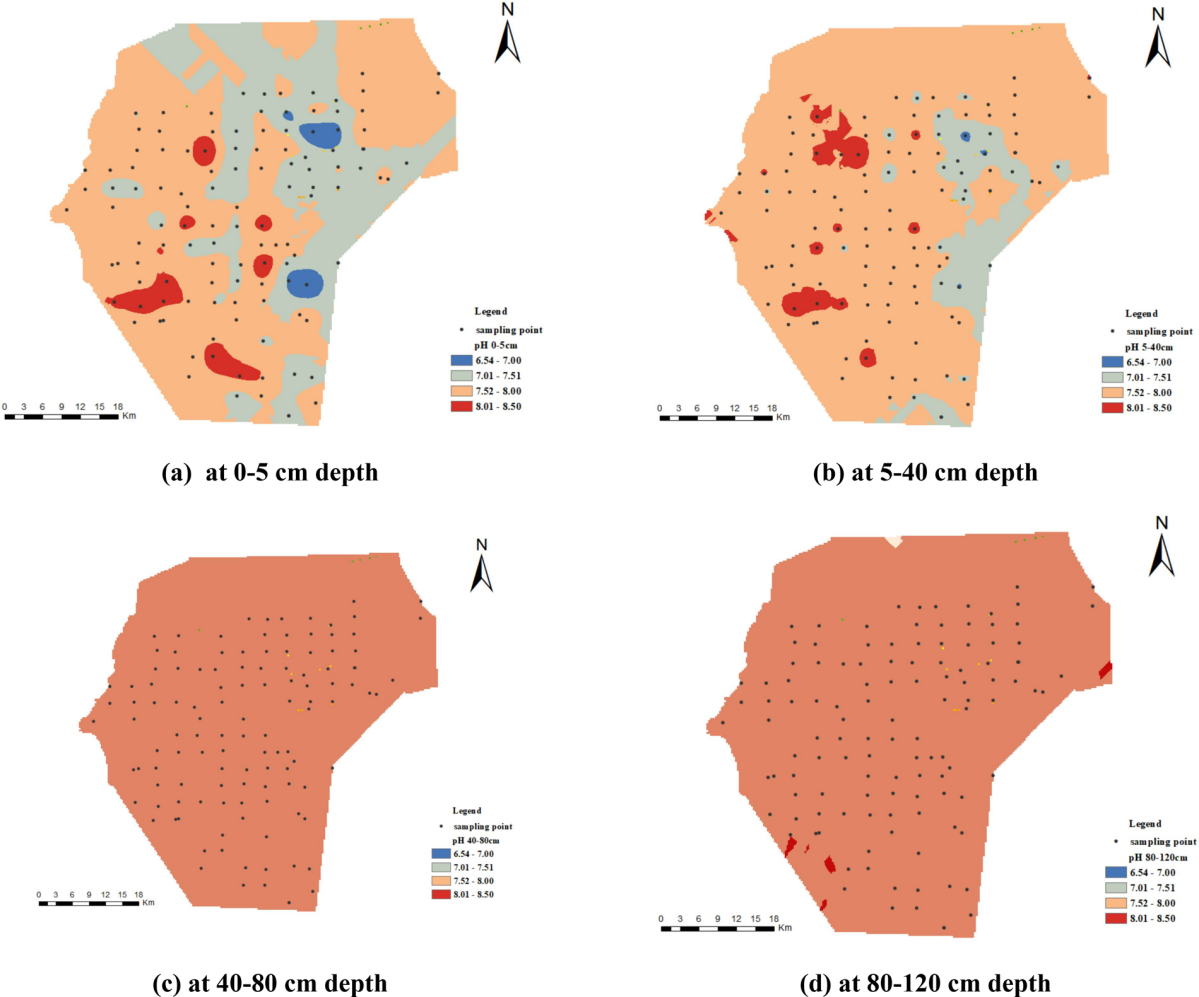
Spatial distribution of pH. (**a**) represents pH at 0-5 cm depth, (**b**) represents pH at 5-40 cm depth, (**c**) represents pH at 40-80 cm depth, and (**d**) represents pH at 80-120 cm depth.

Figure 5 shows that soil pH in the study area ranges from 6.54 to 8.50, indicating a range from weakly acidic to weakly alkaline. Vertically, surface soil exhibits considerable pH variation, with the majority of values ranging between 7 and 8. With increasing soil depth, pH gradually increases and stabilizes around 8 to 8.5. Horizontally, the local central-eastern region displays acidic features with pH values below 7, while the southwestern area indicates alkaline tendencies, with some locations exceeding a pH of 8.

**Fig. 6:**
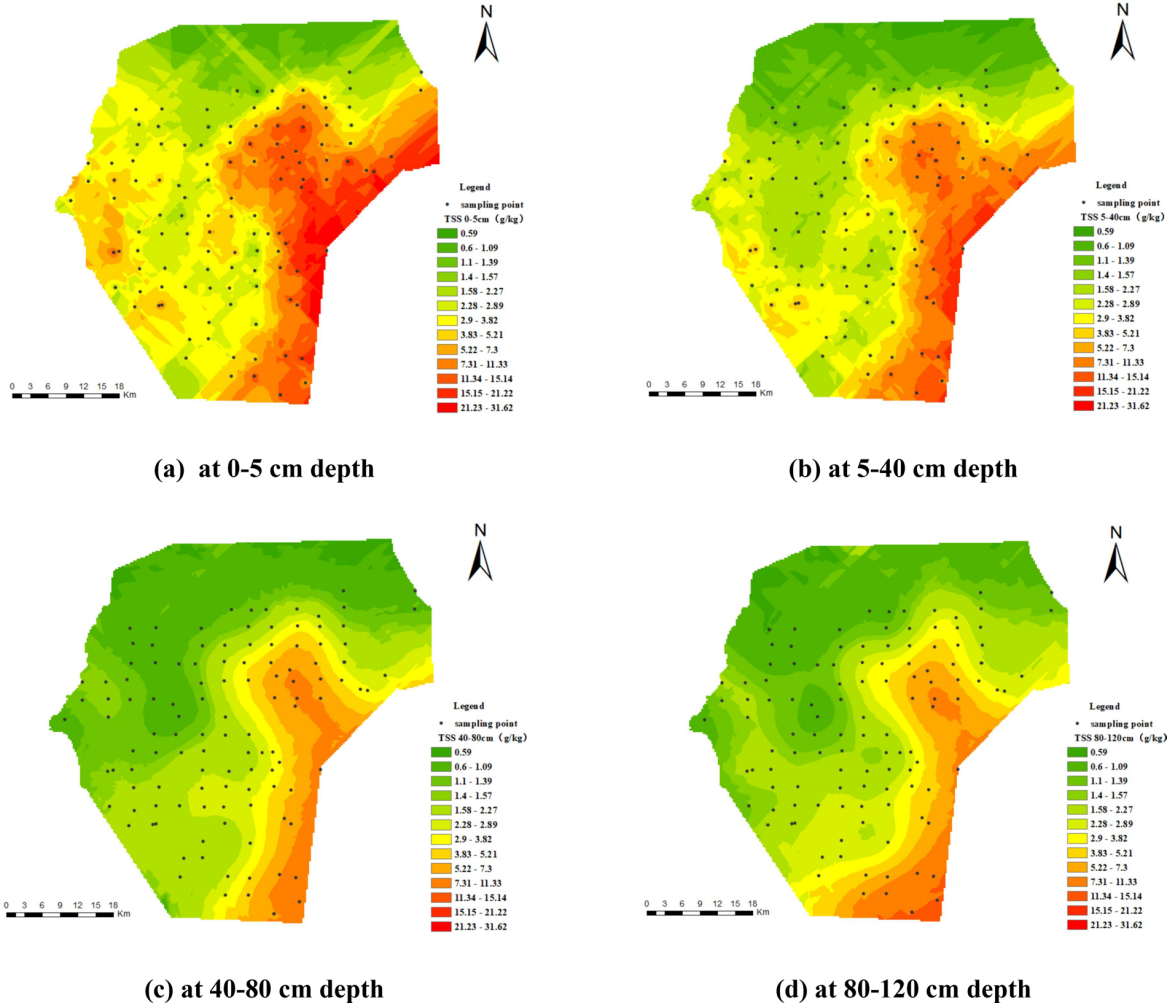
Spatial distribution of soil TSS (g/kg). (**a**) represents TSS at 0–5 cm depth, (**b**) represents TSS at 5–40 cm depth, (**c**) represents TSS at 40–80 cm depth, and (**d**) represents TSS at 80–120 cm depth.

Figure 6 illustrates the spatial distribution of TSS. The spatial distribution of soil TSS remains consistent across different depths, with regions of high soil salt content primarily situated in the southeastern section, while the northwestern area shows lower salt levels. Vertically, soil TSS concentrations are highest in the surface layer, decreasing with depth. Interestingly, the spatial distribution of soil TSS at various depths is relative with that of soil moisture content.

**Fig. 7:**
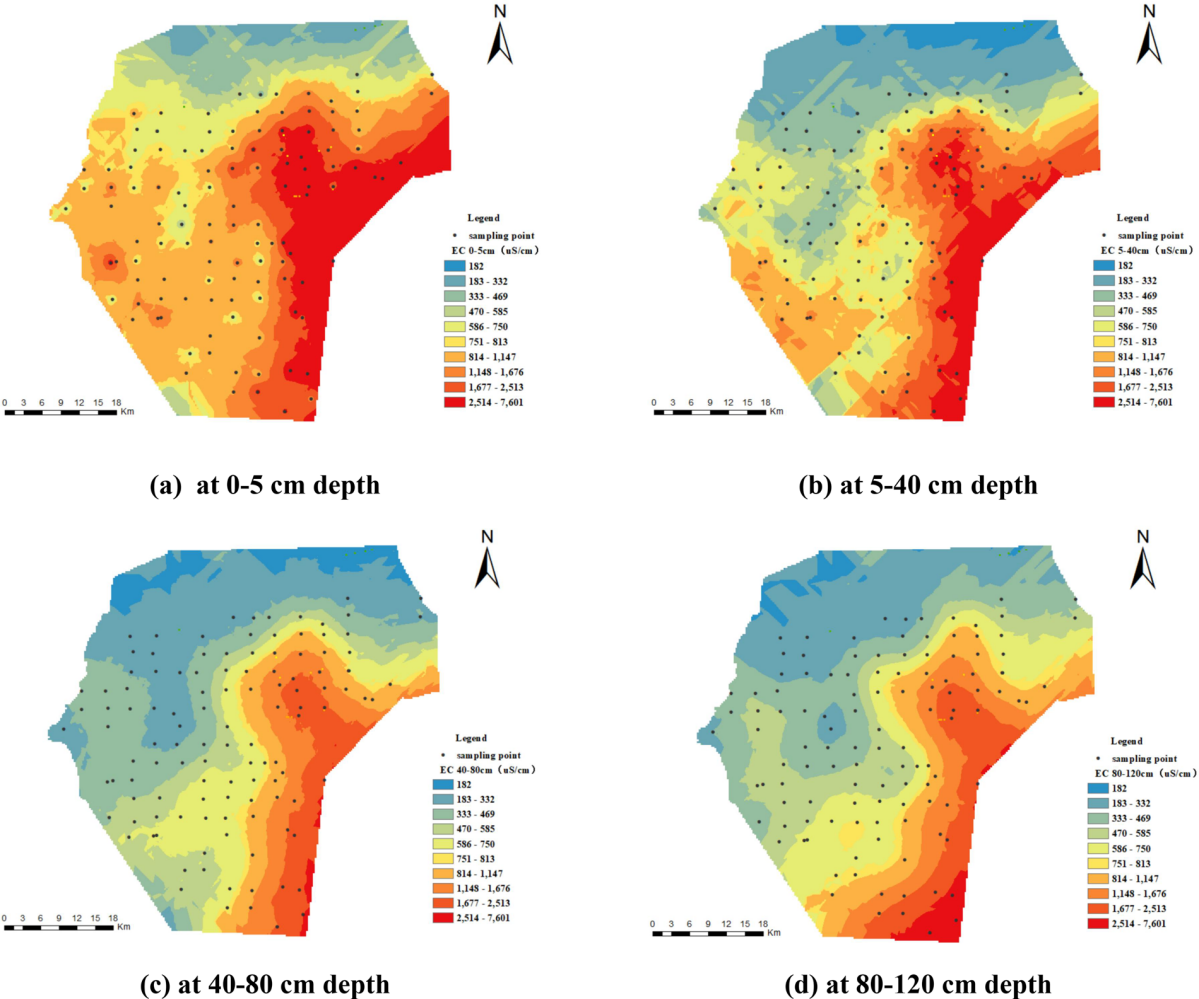
Spatial distribution of electrical conductivity (EC) (uS/cm). (**a**) represents EC at 0–5 cm depth, (**b**) represents EC at 5–40 cm depth, (**c**) represents EC at 40–80 cm depth, and (**d**) represents EC at 80–120 cm depth.

Figure 7 shows the spatial distribution of soil EC, closely corresponding to that of the TSS. A clear trend of gradual increase is evident from the northwest to the southeast. Localized areas of heightened conductivity are notably pronounced in the southeastern and southern parts of the area. Vertically, EC decreases gradually with depth, mirroring the pattern observed in TSS.

**Fig. 8:**
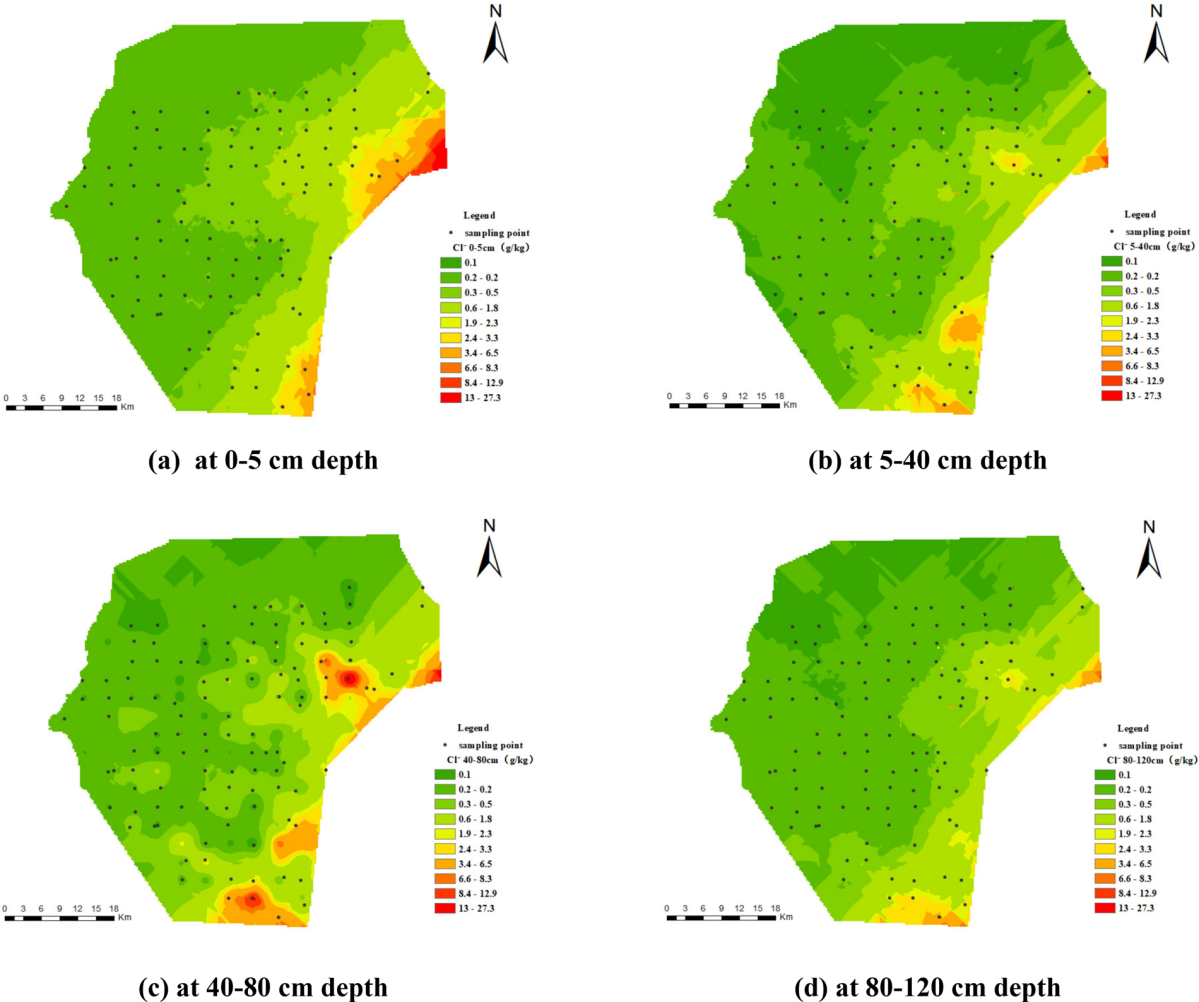
Spatial Distribution of Cl⁻ (g/kg). (**a**) represents Cl⁻ at 0–5 cm depth, (**b**) represents Cl⁻ at 5–40 cm depth, (**c**) represents Cl⁻ at 40–80 cm depth, and (**d**) represents Cl⁻ at 80–120 cm depth.

Figure 8 depicts the spatial distribution of soil chloride ions (Cl⁻). The data indicate that chloride ion concentrations in the majority of areas are below 1.0 g/kg, with a gradual increase from the northwest to the southeast. The ions are mainly clustered in the surface 0–5 cm layer, with concentrations decreasing gradually with depth. Nonetheless, notable localized enrichment is noted in the middle and lower soil layers (at depths of 40–80 cm), especially in the southern and eastern regions of the research area.

**Fig. 9:**
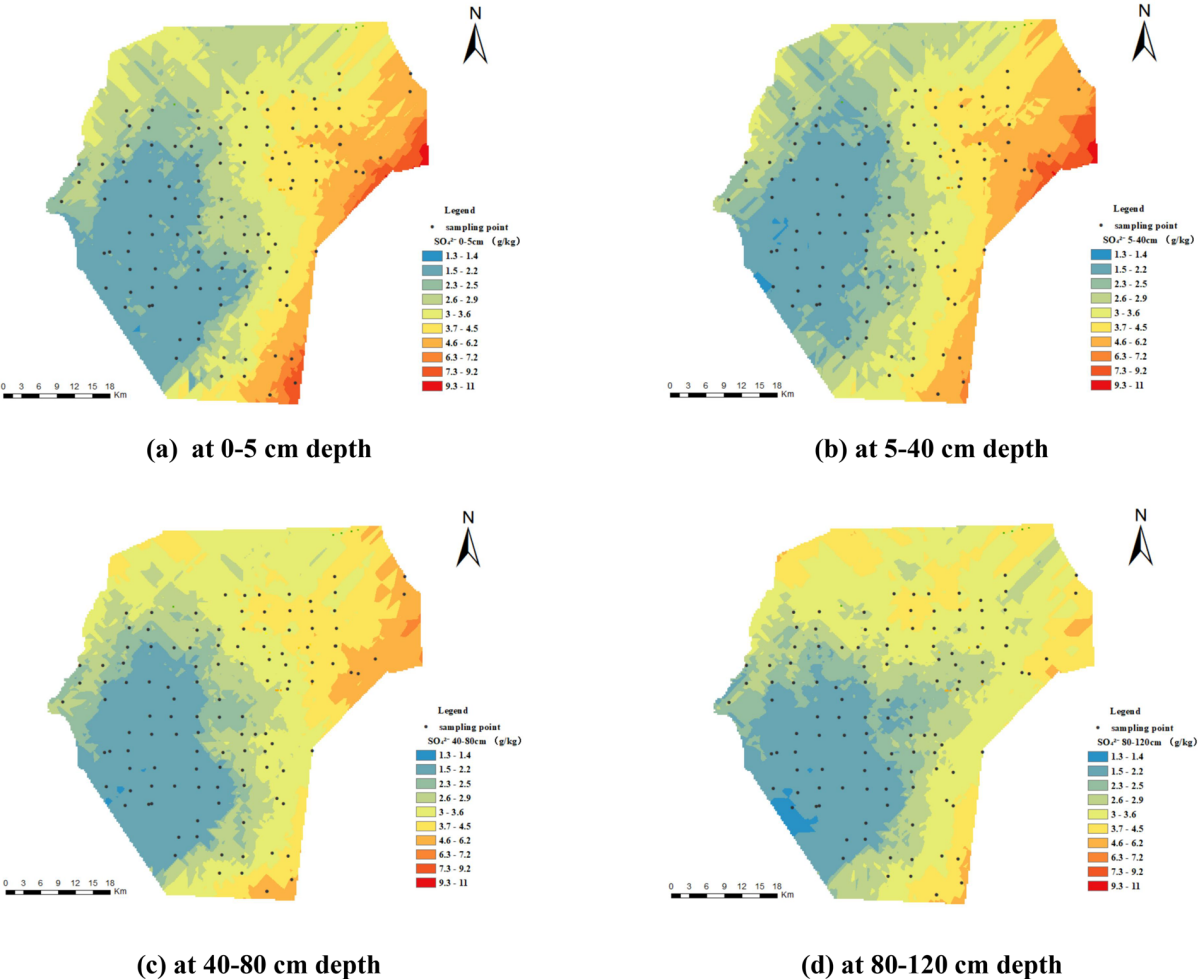
Spatial Distribution of SO₄²⁻ (g/kg). (**a**) represents SO₄²⁻ at 0–5 cm depth, (**b**) represents SO₄²⁻ at 5–40 cm depth, (**c**) represents SO₄²⁻ at 40–80 cm depth, and (**d**) represents SO₄²⁻ at 80–120 cm depth.

Figure 9 depicts the spatial distribution of sulfate ions (SO₄²⁻) in the study area. The concentration of SO₄²⁻ exceeded 1 g/kg throughout the area, surpassing that of Cl^-^. However, the spatial distribution of SO₄²⁻ differs slightly from Cl^-^, with lower levels in the southwestern region and localized aggregation in the central and eastern areas, especially within the 0–40 cm depth range. Additionally, the content of SO₄²⁻ decreases vertically with depth.

## Technical Validation

The quality of the data is ensured on three levers, including data calibration, sample data screening and data analysis.

### Data calibration

#### Systematic error control

Throughout the entire process of this dataset—from sample point selection and field sampling to laboratory analysis—experimental specifications are rigorously adhered to. During the arrangement of sampling points, an equal-interval layout with minimal errors is implemented. Simultaneously, a systematic sampling specification plan is established, and technical training is provided to personnel involved in field sampling to minimize human error. Relevant information, including the longitude, latitude, altitude, temperature, humidity of the sampling points, as well as the type of crops planted, land type, and recent irrigation activities, is recorded in real time.

Soil samples were excavated on-site and immediately placed into sample bags, from which air was excluded prior to sealing. The sealed bags were then wrapped in waterproof cloth to minimize moisture loss during transport to the laboratory. Within three days, the moisture content test was conducted first, followed by the assessment of other indicators. The entire laboratory testing process strictly adheres to the relevant standards, and parallel tests were performed to verify data accuracy in a timely manner. Following the determination of each indicator, the data were promptly entered into the computer, checked for outliers, and additional parallel tests were conducted as necessary. The finalized dataset underwent an initial self-check by laboratory personnel before submission to experts for final review and revision, ensuring the accuracy and validity of the data.

#### Data accuracy

**Fig. 10:**
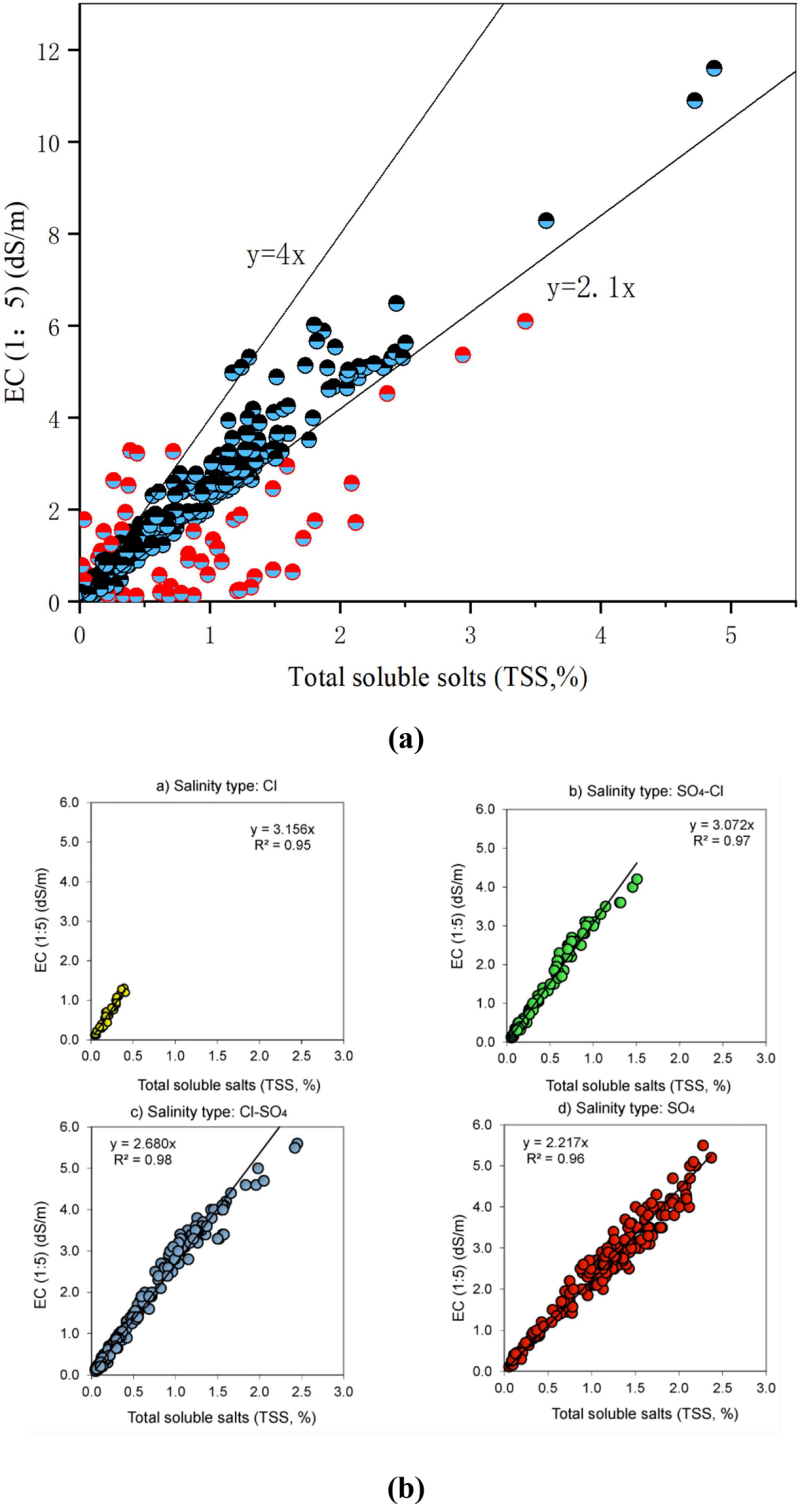
Relationship between electrical conductivity (EC) and total soluble solids (TSS). (**a**) presents the relationship between electrical conductivity (EC) and total suspended solids (TSS) for all test samples collected during this study. In this diagram, red dots represent the rejection points of the samples, while blue dots indicate the qualified retention points. (**b**) presents research conducted by Amin I. Ismayilov *et al*.^26^ on the relationship between various soil salt types’ EC (measured at a soil-to-water ratio of 1:5) and TSS during spring in the semi-arid and arid Kur-Araz Basin of Azerbaijan.

To evaluate the accuracy and reliability of the test data, an analysis examined the relationship between the electrical conductivity (EC) of the leachate and total suspended solids (TSS). The experimental data indicated that the predominant salt ions in the soil of this study area were chloride and sulfate ions. As illustrated in Fig. 10, the slope of the linear relationship between EC (1:5) and TSS (%) at most soil sampling points ranged from 2.1 to 4.0. This outcome is fundamentally consistent with the conclusions drawn by Amin I. Ismayilov *et al*.^26^, who investigated the relationship between EC (soil-to-water 1:5) and TSS across various soil salt types in the Kur–Araz basin of Azerbaijan, a semi-arid and arid region during the spring period. Their findings indicated that the slope of the relationship between EC (1:5) and TSS (%) varied from 2.217 to 3.156 across four distinct salt types: chloride salts, sulfate-chloride salts, chloride-sulfate salts, and sulfate salts.

#### Parallel tests

Due to the large volume of test data, conducting all parallel experiments proves challenging. To minimize experimental error, parallel tests were performed on soil samples collected from 32 randomly selected soil sampling points. The two parallel test values were designated as *X*₁ and *X*₂, allowing for the calculation of the absolute difference (d) and the relative deviation (RD) of the parallel samples.

3 $${\rm{d}}=|{X}_{1}-{X}_{2}|$$

4 $$RD( \% )=\frac{{X}_{1}-{X}_{2}}{{X}_{1}+{X}_{2}}\times 100 \% $$

Table 3 outlines the provisions on parallel sample accuracy in NY/T 1121.02-2016 on pH and in NY/T 1121.16-2016 on TSS. Error analysis of pH and TSS in parallel samples is presented in Table 4, and to determine whether the accuracy requirements are met. The results show that the parallel sample accuracy is basically satisfied.

**Table 3:** The provisions on parallel sample accuracy in NY/T 1121.

Variable	Standard	Range of TSS	Allowable relative deviation RD(%)
TSS (g/kg)	NY/T 1121.16-2016	<0.5	<20
		0.5–2	15–10
		2–5	10–5
		>5	<5
**Variable**	**Standard**	**Range of pH**	**Allowable absolute difference** ***d***
pH	NY/T 1121.2-2016	≤7	≤0.1
		å 7	≤0.2

**Table 4:** Error Analysis of Typical Parameters of Parallel Samples.

Soil Sampling Number(SSN)	pH					TSS				
	1#	2#	$\bar{{X}}$	*d*	Does it meet the accuracy requirements?	1#	2#	$\bar{{X}}$	RD(%)	Does it meet the accuracy requirements?
2-100	7.37	7.47	7.42	0.1	Yes	14.9	13.4	14.15	5.30	No
7-80	7.94	7.92	7.93	0.02	Yes	2.7	2.5	2.60	3.85	Yes
9-5	7.67	7.67	7.69	0	Yes	2.5	2.5	2.50	0.00	Yes
10-5	8.25	8.20	8.23	0.05	Yes	2.3	2.8	2.55	9.80	Yes
13-100	7.75	7.88	7.82	0.13	Yes	8.2	9.8	9.00	8.89	No
31-5	7.59	7.56	7.58	0.03	Yes	3.2	3.6	3.40	5.88	Yes
32-40	7.96	7.95	7.96	0.01	Yes	1.6	1.4	1.50	6.67	Yes
35-100	7.61	7.62	7.62	0.01	Yes	1.0	0.9	0.95	5.26	Yes
38-5	8.06	7.93	8.00	0.13	Yes	4.1	4.3	4.20	2.38	Yes
40-40	7.88	7.80	7.84	0.08	Yes	1.6	1.6	1.60	0.00	Yes
41-5	8.35	8.33	8.34	0.02	Yes	2.9	2.9	2.90	0.00	Yes
42-100	8.21	8.17	8.19	0.04	Yes	0.4	0.4	0.40	0.00	Yes
43-40	8.21	8.14	8.18	0.07	Yes	1.6	1.7	1.65	3.03	Yes
44-60	7.94	7.64	7.79	0.3	No	5.4	6.1	5.75	6.09	No
65-20	7.71	7.71	7.71	0	Yes	2.0	2.1	2.05	2.44	Yes
66-5	7.46	7.49	7.48	0.03	Yes	7.1	7.2	7.15	0.70	Yes
68-40	7.71	7.78	7.75	0.07	Yes	1.8	1.6	1.70	5.88	Yes
73-5	7.74	7.73	7.74	0.01	Yes	1.3	1.2	1.25	4.00	Yes
76-60	7.61	7.58	7.60	0.03	Yes	3.1	3.4	3.25	4.62	Yes
78-80	7.73	7.78	7.76	0.05	Yes	0.5	0.6	0.55	9.09	Yes
85-60	7.54	7.55	7.55	0.01	Yes	0.7	0.6	0.65	7.69	Yes
86-5	8.22	8.18	8.20	0.04	Yes	0.7	0.7	0.70	0.00	Yes
90-100	8.19	8.04	8.12	0.15	Yes	1.8	1.7	1.75	2.86	Yes
92-60	8.01	7.96	7.99	0.05	Yes	0.5	0.5	0.50	0.00	Yes
110-5	7.73	7.76	7.75	0.03	Yes	7.0	7.5	7.25	3.45	Yes
111-120	8.21	8.29	8.25	0.08	Yes	0.6	0.7	0.65	7.69	Yes
115-120	8.14	8.10	8.12	0.04	Yes	5.3	5.7	5.50	3.64	Yes
142-20	8.14	8.13	8.14	0.01	Yes	1.5	1.4	1.45	3.45	Yes
162-120	7.52	7.49	7.51	0.03	Yes	2.8	3.2	3.00	6.67	Yes
167-100	8.04	8.01	8.03	0.03	Yes	0.8	0.8	0.80	0.00	Yes
169-40	7.80	7.80	7.80	0	Yes	0.5	0.8	0.65	23.08	No
175-5	7.54	7.52	7.53	0.02	Yes	2.0	2.0	2.00	0.00	Yes

### Sample data screening

**Fig. 11:**
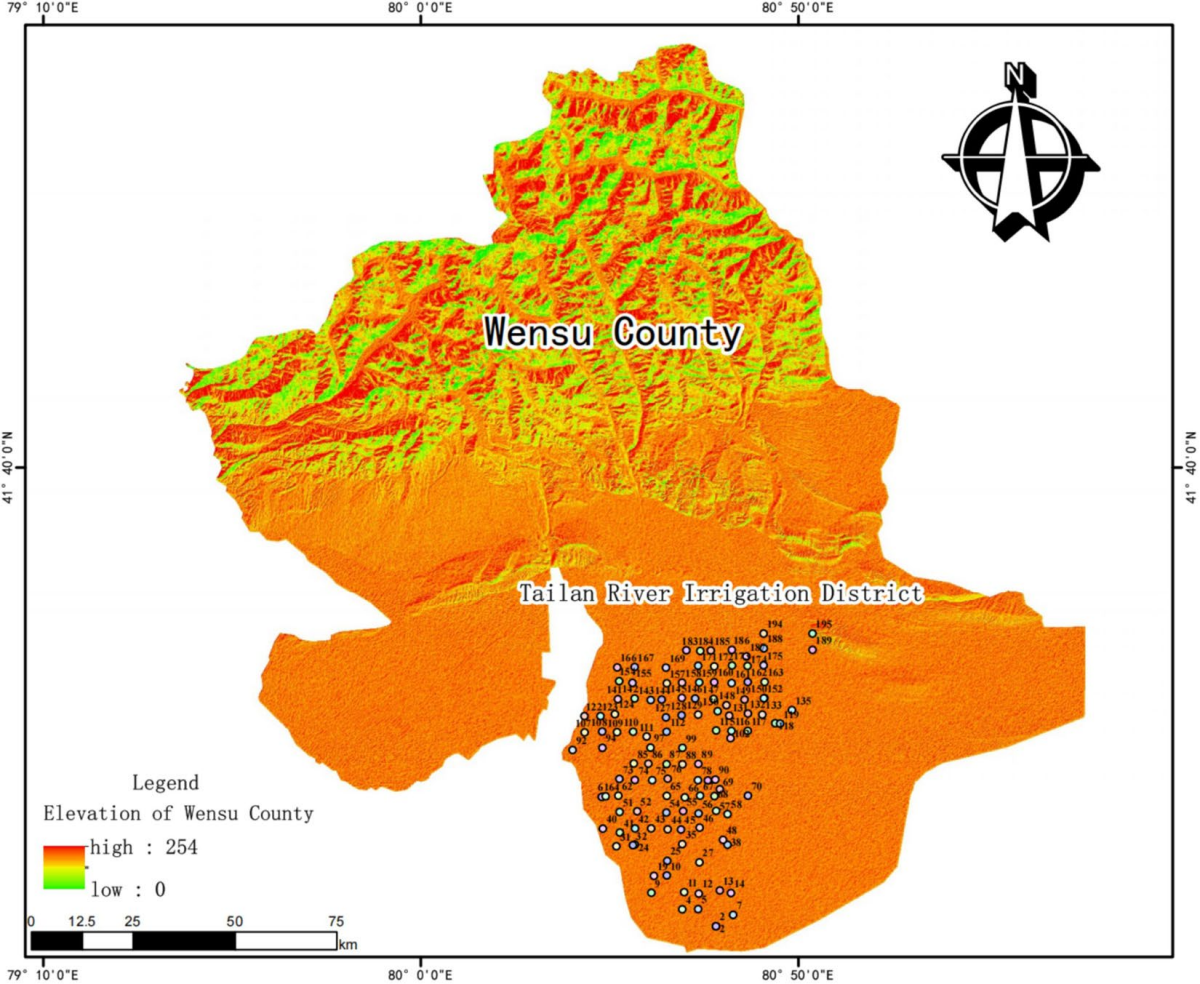
Schematic diagram of valid sampling point distribution after data calibration. It presents the distribution of the 118 valid sampling points.

A total of 164 sampling points were established on-site, with each point consisting of 7 soil samples collected at depths ranging from 0 to 120 cm. If the soil was extremely dry, the sampling depth was limited to 60-80 cm instead of 120 cm. This approach resulted in a total of 1,148 samples. After verification using the soil electrical conductivity (EC) and total dissolved solids (TSS) proportional relationship, along with parallel tests, a total of 118 qualified sampling points and 807 valid data records were ultimately identified. Figure 11 illustrates the distribution of these valid sampling points.

These 807 valid data represent 70.3% of the total soil samples and form the dataset of soil physical and chemical properties in the Tailan River Irrigation District.

### Data analysis

**Fig. 12:**
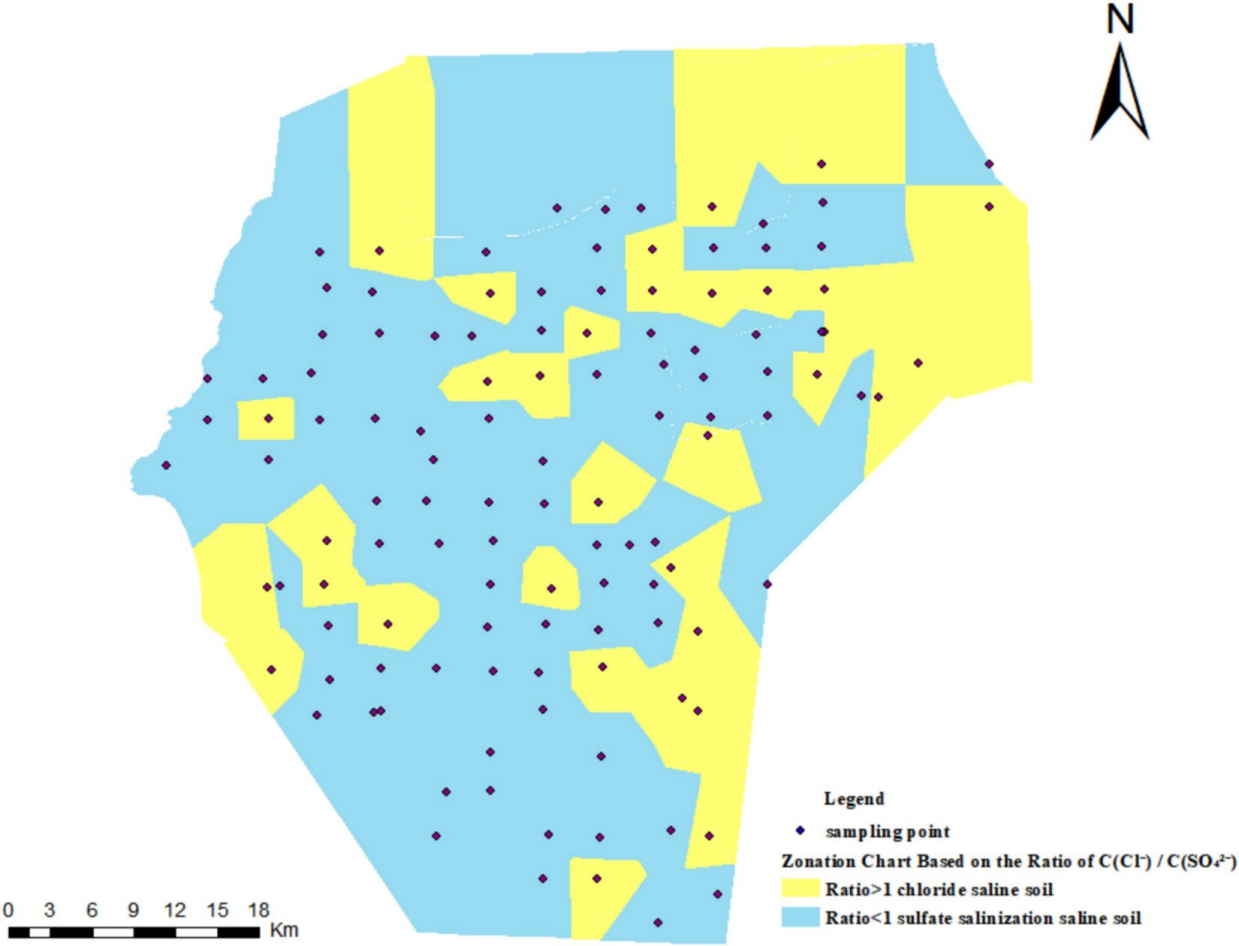
Salinization Zonation chart based on the ratio c(Cl⁻)/c(SO₄²⁻). It presents that the yellow region represents the condition where c(Cl^-^) > c(SO_4_^2-^), which is designated as the chloride saline soil zone. Conversely, the blue region indicates the condition where c(SO_4_^2-^) > c(Cl^-^), identified as the sulfate saline soil zone.

This dataset provides crucial support for a comprehensive understanding of soil properties in irrigation districts, improving management efficiency. Through analyzing the spatial distribution characteristics of moisture content (MC), electrical conductivity (EC), pH, total suspended solids (TSS), and eight different ions, it enables the swift classification of salinity levels, identification of salinization risk zones, prevention of crop damage due to salt, and the development of targeted enhancement strategies. According to the provisions of the soil classification system in the third national soil census of China, saline soils in arid regions are classified based on their relative salt content, with detailed classification criteria outlined in Table 5. Test data indicated low concentrations of CO_3_^2-^ and HCO_3_^-^ in the soil of the Tailan River Irrigation District. The salinization zoning map of Tailan River Irrigation District was drawn according to the classification method in Table 5, as shown in Fig. 12. The distribution pattern of salinization is basically consistent within the depth range of 0-120 cm, indicating that the area is mainly composed of sulfate saline soil, followed by chloride saline soil.

**Table 5:** Classification and grading standard of saline soil.

Classification	TSS (g/kg)	Salinization Grading
chloride saline soil (Cl^-^ + SO_4_^2-^ > CO_3_^2-^ + HCO_3_^-^, and Cl^-^ > SO_4_^2-^)	1 ≤ TSS < 4	Slight salinization
	4 ≤ TSS < 8	Moderate salinization
	8 ≤ TSS < 12	Severe salinization
sulfate salinization saline soil (Cl^-^ + SO_4_^2-^ > CO_3_^2-^ + HCO_3_^-^, and SO_4_^2-^ > Cl^-^)	2 ≤ TSS < 6	Slight salinization
	6 ≤ TSS < 10	Moderate salinization
	10 ≤ TSS < 16	Severe salinization
carbonate salinized saline soil (CO_3_^2-^ + HCO_3_^-^ > Cl^-^ + SO_4_^2-^)	1 ≤ TSS < 3	Slight salinization
	3 ≤ TSS < 5	Moderate salinization
	5 ≤ TSS < 7	Severe salinization

The classification of saline soil is helpful to guide the implementation of water-conserving irrigation techniques, utilization of soil amendments, and cultivation of salt-tolerant crops. When integrated with groundwater levels and soil texture information, the dataset enhances the design of drainage systems. Moreover, it supports the creation of water-salt movement models and the establishment of ecological restoration plans. This dataset is relevant across interdisciplinary research fields such as agriculture, hydrology, and ecology, facilitating the shift from broad management to precise governance in irrigation districts. It also lays the groundwork for regional policy formulation and collaborative scientific research, serving as a critical asset in addressing salinity issues and promoting sustainable land use.

## Data Availability

The dataset is freely available through Figshare’s public link at: 10.6084/m9.figshare.30084454^25^.

## Code availability

No custom code was used in this study.
